# 4507 Developing and implementing a Principal Investigator (PI) primer to improve the conduct of human research at the University of Minnesota

**DOI:** 10.1017/cts.2020.206

**Published:** 2020-07-29

**Authors:** Jessica Wright, Jennifer Maas, Megan Hoffman

**Affiliations:** 1 University of Minnesota CTSI

## Abstract

OBJECTIVES/GOALS:

- Assess the institutional and individual training needs and gaps in the conduct of human research for PIs at the University of Minnesota.
- Define the training program’s learning objectives.
- Develop and implement an in-person training session that addresses the gaps.

METHODS/STUDY POPULATION:

- Establish a planning committee
- Identify required and optional training that is already available for PIs, then determine gaps
- Understand research training needs based on conversations with departmental and human research protection program leaders.
- Develop learning objectives and curriculum based on Federal and Local regulations, guidelines, and policies.
- Establish a feedback loop regarding research compliance with the HRPP, to assess trends and ensure continuous improvement.
- Evaluate the training program’s participants using confidence and satisfaction measures.

RESULTS/ANTICIPATED RESULTS: Developed and piloted a 90-minute in-person training program entitled “PI Primer” with the goals of:

- Increasing awareness and knowledge of the role and responsibilities of the Principal Investigator (PIs) according to the International Harmonization for Good Clinical Practice (ICH-GCP), Federal Regulations (FDA, DHHS, ect.), and University of Minnesota Policies.
- Identifying root causes for receiving an FDA 483 (inspection findings).
- Addressing and preventing common inspection findings (CAPA).
- Describe individual and institutional conflict of interest (COI), and identify the key steps necessary to manage COIs.

DISCUSSION/SIGNIFICANCE OF IMPACT: OHRP’s guidance on the “Responsibilities of Investigators” states that it is the Institution’s responsibility to provide human research training on a wide variety of topics to ensure the ethical conduct of research and protection of participants. PI Primer provides an in-person forum for investigators to build upon required responsible conduct of research and good clinical practice training to be able to apply the role and responsibilities of a PI to their own research. PI Primer also establishes a network of PIs in order to enhance connectivity and shared learning.

